# Relationship Among Macronutrients, Dietary Components, and Objective Sleep Variables Measured by Smartphone Apps: Real-World Cross-Sectional Study

**DOI:** 10.2196/64749

**Published:** 2025-01-30

**Authors:** Jaehoon Seol, Masao Iwagami, Megane Christiane Tawylum Kayamare, Masashi Yanagisawa

**Affiliations:** 1 International Institute for Integrative Sleep Medicine (WPI-IIIS) University of Tsukuba Tsukuba, Ibaraki Japan; 2 Institute of Health and Sport Sciences University of Tsukuba Tsukuba, Ibaraki Japan; 3 Department of Frailty Research Center for Gerontology and Social Science National Center for Geriatrics and Gerontology Obu, Aichi Japan; 4 Research Team for Promoting Independence and Mental Health Tokyo Metropolitan Institute of Gerontology Tokyo Japan; 5 Institute of Medicine University of Tsukuba Tsukuba, Ibaraki Japan; 6 PhD Program in Humanics University of Tsukuba Tsukuba, Ibaraki Japan; 7 S’UIMIN Inc Shibuya, Tokyo Japan; 8 Life Science Center for Survival Dynamics (TARA) University of Tsukuba Tsukuba, Ibaraki Japan; 9 R&D Center for Frontiers of Mirai in Policy and Technology (F-MIRAI) University of Tsukuba Tsukuba, Ibaraki Japan; 10 Department of Molecular Genetics University of Texas Southwestern Medical Center Dallas, TX United States

**Keywords:** sleep quality, dietary health, unsaturated fatty acids, dietary fiber intake, sodium-to-potassium ratio, compositional data analysis, sleep, smartphone, application

## Abstract

**Background:**

Few studies have explored the relationship between macronutrient intake and sleep outcomes using daily data from mobile apps.

**Objective:**

This cross-sectional study aimed to examine the associations between macronutrients, dietary components, and sleep parameters, considering their interdependencies.

**Methods:**

We analyzed data from 4825 users of the Pokémon Sleep and Asken smartphone apps, each used for at least 7 days to record objective sleep parameters and dietary components, respectively. Multivariable regression explored the associations between quartiles of macronutrients (protein; carbohydrate; and total fat, including saturated, monounsaturated, and polyunsaturated fats), dietary components (sodium, potassium, dietary fiber, and sodium-to-potassium ratio), and sleep variables (total sleep time [TST], sleep latency [SL], and percentage of wakefulness after sleep onset [%WASO]). The lowest intake group was the reference. Compositional data analysis accounted for macronutrient interdependencies. Models were adjusted for age, sex, and BMI.

**Results:**

Greater protein intake was associated with longer TST in the third (+0.17, 95% CI 0.09-0.26 h) and fourth (+0.18, 95% CI 0.09-0.27 h) quartiles. In contrast, greater fat intake was linked to shorter TST in the third (–0.11, 95% CI –0.20 to –0.27 h) and fourth (–0.16, 95% CI –0.25 to –0.07 h) quartiles. Greater carbohydrate intake was associated with shorter %WASO in the third (–0.82%, 95% CI –1.37% to –0.26%) and fourth (–0.57%, 95% CI –1.13% to –0.01%) quartiles, while greater fat intake was linked to longer %WASO in the fourth quartile (+0.62%, 95% CI 0.06%-1.18%). Dietary fiber intake correlated with longer TST and shorter SL. A greater sodium-to-potassium ratio was associated with shorter TST in the third (–0.11, 95% CI –0.20 to –0.02 h) and fourth (–0.19, 95% CI –0.28 to –0.10 h) quartiles; longer SL in the second (+1.03, 95% CI 0.08-1.98 min) and fourth (+1.50, 95% CI 0.53-2.47 min) quartiles; and longer %WASO in the fourth quartile (0.71%, 95% CI 0.15%-1.28%). Compositional data analysis, involving 6% changes in macronutrient proportions, showed that greater protein intake was associated with an elevated TST (+0.27, 95% CI 0.18-0.35 h), while greater monounsaturated fat intake was associated with a longer SL (+4.6, 95% CI 1.93-7.34 min) and a larger %WASO (+2.2%, 95% CI 0.63%-3.78%). In contrast, greater polyunsaturated fat intake was associated with a reduced TST (–0.22, 95% CI –0.39 to –0.05 h), a shorter SL (–4.7, 95% CI to 6.58 to –2.86 min), and a shorter %WASO (+2.0%, 95% CI –3.08% to –0.92%).

**Conclusions:**

Greater protein and fiber intake were associated with longer TST, while greater fat intake and sodium-to-potassium ratios were linked to shorter TST and longer WASO. Increasing protein intake in place of other nutrients was associated with longer TST, while higher polyunsaturated fat intake improved SL and reduced WASO.

## Introduction

Dietary intake patterns vary widely depending on the lifestyle. Brain imaging reveals that these patterns are associated with mental health, cognitive function, blood and metabolic biomarkers, overall brain structure, and white matter integrity [1]. Diet and sleep are closely linked [2]. A systematic review partially revealed that overeating, especially meals high in fat, can extend sleep latency (SL), increase wakefulness after sleep onset (WASO), and ultimately reduce total sleep time (TST) [3-5].

Dietary intake and sleep are known to have a bidirectional relationship [2]. The intake of healthy foods is associated with improved sleep quality, whereas consuming processed foods and foods high in free sugars has been shown to deteriorate sleep quality [6]. Several hypotheses have been proposed to explain this relationship [7-9]. One hypothesis suggests that an increased dietary intake of tryptophan can promote the production of serotonin and melatonin, potentially leading to improved sleep quality [7]. Additionally, sleep deprivation can decrease leptin levels and increase ghrelin levels, a hormone that stimulates appetite, and may trigger overeating, especially at night, creating a negative spiral [7-9].

The importance of assessment methods in examining the relationship between diet and sleep has been emphasized in numerous studies [2,3,10]. Subjective methods, such as dietary diaries, 24-hour recalls, and food frequency questionnaires, rely heavily on the participants’ memory and can be prone to recall bias. In contrast, objective methods, such as digital apps that record dietary intake in real time, can reduce bias; however, they still face challenges related to technology use and data accuracy [11]. Furthermore, automated systems have been introduced to eliminate recording and subjective biases, allowing users to register menus or scan the barcodes of commercially available products, which automatically calculates the nutritional components [12]. Similarly, sleep assessments also face challenges. Subjective sleep assessments, such as sleep diaries or self-reported questionnaires, are prone to recall bias, sleep-state misperception, and underreporting. Objective assessments, including polysomnography, portable electroencephalogram devices, and wearable devices such as accelerometers, provide more accurate data but are often costly, require technical expertise, or can be intrusive for users [11,13-15]. Each method has its own set of limitations, making it important to carefully choose the appropriate assessment method based on the research context.

Observational studies investigating the relationship between diet and sleep often rely on subjective assessments of nutrition and sleep, with many highlighting the potential for recall bias [2,3]. Many studies have focused on the intake of individual nutrients, such as specific proteins, carbohydrates, fatty acids, and vitamins [16-20]. Specifically, increased protein intake has been reported to improve subjective sleep quality [16,17], while carbohydrate intake affects the sleep stages, SL, and WASO; however, further research is warranted on its long-term impact [21]. Imbalances in polyunsaturated fatty acids have been linked to a higher prevalence of sleep disorders and shorter TST [19,20]. Additionally, vitamin D deficiency has been associated with sleep disorders and shorter TST [18]. However, macronutrients that contribute to the total energy intake (such as carbohydrates, proteins, and fats, including saturated and unsaturated fatty acids) are interdependent and interact with each other [22]. Although recent research has considered these interdependencies and their relationship with health status [22-24], research on how these interdependencies affect sleep is limited. In particular, these methods, which consider the interdependencies of nutrient components, are expected to attract more attention in the future but have not yet been fully explored [25].

Understanding these complex relationships is crucial before transitioning to intervention studies, as it provides a foundational understanding of how nutrients work together to influence health outcomes. Therefore, an approach that accounts for the interdependent relationships between nutrients is necessary. This cross-sectional study using real-world data from smartphone apps aimed to examine the hypothesis that there are relationships among macronutrients, dietary components, and sleep parameters. We also investigated the relationships between sleep and macronutrients while considering their interdependencies using compositional data analysis.

## Methods

### Study Design and Participants

This retrospective, cross-sectional study used data from 6052 users who consented to participate between January 19 and 31, 2024. During this period, in-app messages were sent to all the users of the Asken app (Asken Inc), which is designed for dietary management and recording, requesting permission to use their stored data. The goal of the Asken app was to gather detailed information on dietary intake, enabling us to assess the participants’ nutritional habits.

At the same time, users who also used Pokémon Sleep (The Pokémon Company), a sleep-tracking app, were asked to provide their user IDs in their electronic informed consent. This linking of data was crucial for analyzing the relationship between the objective sleep data collected from the Pokémon Sleep app and the dietary data from the Asken app.

### Ethical Considerations

This study was conducted in accordance with the Declaration of Helsinki and approved by the Institutional Review Board of Sapporo Yurinokai Hospital, Japan (approval 030). Informed consent was obtained from all participants involved in the study. Participants used both apps concurrently from July 2023 to January 2024. Furthermore, data were extracted only from the overlapping period between the start and end dates of both apps, and average values were considered representative of each participant’s typical sleep habits and nutritional intake status. To ensure participant privacy and confidentiality, all data were deidentified before analysis, and no personally identifiable information was included in the dataset. The dataset was securely stored and accessible only to authorized researchers. Participants were not provided with monetary compensation for their participation in the study. However, they were fully informed about the study's purpose and significance, and their voluntary participation was obtained based on informed consent.

### Dietary Intake Patterns Assessment

We calculated dietary intake patterns using the Asken smartphone app. Participants logged their meals by selecting food items and portion sizes from a database containing over 100,000 menu items. Participants entered the details of each meal into the app. For commercial products, the nutritional information was automatically recorded by scanning the barcode. The nutritional values were calculated based on the *2020 Standard Tables of Food Composition in Japan (eighth edition)* [26]. The recording and calculation methods used by the participants are detailed in a previous study [12]. The Asken dietary recording app and traditional paper-based questionnaires have been reported to have a reasonably good correlation coefficient of 0.80 for nutrient intake [12,27,28].

In this retrospective, cross-sectional study, data were only included from days when participants reported consuming all three meals, as it was not possible to distinguish between missed recordings and actual skipped meals. Asken allows for the calculation of various macronutrients and dietary components, including total energy, protein, total fat (including saturated, monounsaturated, and polyunsaturated fats), carbohydrate, sodium, potassium, calcium, magnesium, phosphorus, iron, zinc, copper, manganese, vitamin A, vitamin D, vitamin E, vitamin K, vitamin B1, vitamin B2, niacin, vitamin B6, vitamin B12, folate, pantothenic acid, vitamin C, cholesterol, dietary fiber, and alcohol intake.

Based on previous research [29,30], we selected and analyzed the following components reportedly associated with sleep: total energy, protein, total fat (including saturated, monounsaturated, and polyunsaturated fats), carbohydrate, sodium, potassium, and dietary fiber intake, and calculated the sodium-to-potassium ratio. Macronutrients were energy adjusted by calculating the percentage of total energy intake. The absolute daily intake was calculated for other dietary components.

### Sleep Assessment

We calculated the TST, SL, and percentage of WASO (%WASO) derived from the Pokémon Sleep, which uses the Cole-Kripke algorithm for sleep-wake determination [31], as publicly disclosed [32]. Although accelerometer-based sleep determination integrated into smartphones lacks the ability to distinguish sleep stages [33,34], it provides a certain level of accuracy in determining sleep and wakefulness [22,23]. Although there may be a slight underestimation of SL, the overall TST and nocturnal awakenings showed good agreement with traditional actigraphy [34,35]. As previously mentioned, Natale et al [35] used the Cole-Kripke algorithm with smartphone accelerometers. Briefly, sleep-wake determination was performed using accelerometer data with 10-second epochs per minute. The decision was based on a 7-minute window of accelerometer data (from 4 min before to 3 min after the reference point [0 min]). A value greater than 1 indicates wakefulness and a value less than 1 indicates sleep [31]. SL was defined as the time from going to bed (start of the recording) to the first instance of being determined as sleep.

### Statistical Analysis

Descriptive analysis was conducted to summarize the basic characteristics of the participants, including sleep status and dietary nutrient intake. The multivariable regression analysis, isotemporal substitution model, and compositional data analysis were performed using the *lmtest*, *deltacomp*, *Compositions*, *robCompositions*, and *ggplot2* R packages, respectively [25,36-38]. All regression models were adjusted for age, sex, and BMI.

First, multivariable regression analysis using the forced-entry method was used to determine the association among macronutrients, dietary components, and objective sleep variables (TST, SL, and %WASO). We conducted quartile categorization for total energy intake, energy-adjusted macronutrients (protein intake, total fat intake including saturated, monounsaturated, and polyunsaturated fats, and carbohydrate intake), sodium intake, potassium intake, dietary fiber intake, and sodium-to-potassium ratio. All the quartile range information for each variable is presented in Table S1 in Multimedia Appendix 1. Dummy variables were created for each nutrient based on the first quartile.

Second, to investigate the linear relationship between macronutrient intake and sleep parameters, we used an isotemporal substitution model and compositional data analysis [22-24]. In the isotemporal substitution model, when one macronutrient component is replaced with another on a one-to-one basis, the other components remain constant [22,23]. Compositional data analysis, in contrast, considers the overall composition and estimates the changes in sleep variables when the proportion of a specific macronutrient component is increased or decreased within the total composition [25]. Both methods have been used in studies that consider the interdependencies of factors contributing to a total composition of 100% (eg, dietary nutrient intake, 24-h physical/sedentary activity, and TST) [22,23,37,38].

We calculated the macronutrient intake (in grams) by multiplying each intake by its respective calorie contribution (ie, 4 kcal for carbohydrates and protein and 9 kcal for each type of fat), ensuring that the sum equals 1 (ie, total energy intake). Nutrient intake data were transformed into compositional data using an additive log-ratio transformation. Nutrient intake data were transformed into compositional data using an additive log-ratio transformation. This transformation was necessary to account for the relative proportions of macronutrients and facilitate subsequent analyses. The log transformation helps meet the assumptions required for compositional data analysis [25]. The variability in the data, in terms of the variability of each macronutrient intake relative to the variability of other components and the total variance of the whole composition, is described in Table S2 in Multimedia Appendix 1 through a variation matrix within each component. A value close to zero implied that the intake of the two macronutrient components was highly proportional.

The compositional data were then subjected to an isometric log-ratio (ilr) transformation to facilitate the application of the linear regression models. The ilr transformation was conducted using default and customized orthonormal bases for different nutrients. Linear regression models were constructed to evaluate the association between sleep parameters and the ilr-transformed nutrient composition after adjusting for sex, age, and BMI (Table S3 in Multimedia Appendix 1). The mean nutrient composition was calculated, and predictions for sleep parameters were made using a linear regression model. The compositional ratios of macronutrients among the participants are presented in Table S4 in Multimedia Appendix 1. Because of the isotemporal substitution model and compositional data analysis, it was not possible to replace values below the minimum composition. Therefore, in this study, we replaced values exceeding the upper limit of 6% (Table S4 in Multimedia Appendix 1).

To assess the impact of altering the nutrient composition, specific changes (eg, increasing monounsaturated fat and decreasing polyunsaturated fat) were applied, and new predictions were obtained. CIs for the differences in the predicted sleep parameters were computed using the residual standard error, model matrix, and critical value from the *t*-distribution. Hypothesis tests were conducted to examine the significance of the associations between individual nutrients and sleep parameters. Different ilr transformations were applied, each focusing on a specific nutrient (protein, carbohydrate, saturated fat, monounsaturated fat, or polyunsaturated fat), and the corresponding linear models were analyzed for significance.

Ramsey regression equation specification error test confirmed the linearity assumption for all models (TST, SL, and %WASO). Variance inflation factor values ranged from 1.10 to 4.65, indicating no multicollinearity. The Durbin-Watson test showed no autocorrelation in the residuals, and the Breusch-Pagan test confirmed homoscedasticity. Although the Shapiro-Wilk test showed significant *P* values, W-values between 0.996 and 0.997 indicated that the residuals were close to normally distributed.

All descriptive and statistical analyses were performed using R (version 4.4.0; R Foundation for Statistical Computing), with statistical significance assessed at the level of .05.

## Results

**Figure 1:**
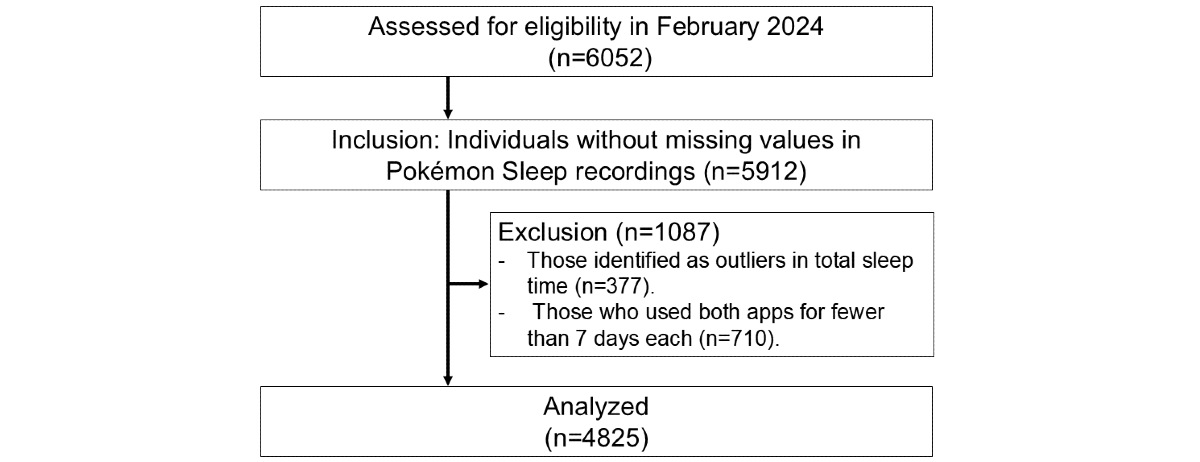
Participant flow chart.

Of the 6052 participants who consented, 140 individuals were excluded from the analysis due to missing sleep records, 377 were excluded due to being outliers in the TST (ie, participants who did not fall within the average SD 3 range), and 710 were excluded because they used both apps for fewer than 7 days each. Therefore, the final analysis included 4825 participants (mean age 36.7, SD 10.4 years; BMI 24.8, SD 4.8 kg/m²; 3938/4825, 81.6% were female; Figure 1 and Table 1).

**Table 1:** Characteristics of participants.

Variables		All participants (n=4825)	Male participants (n=887)	Female participants (n=3938)
**Basic characteristics**				
	Age (years), mean (SD)	36.7 (10.4)	38.4 (10.8)	36.3 (10.2)
	BMI (kg/m^2^), mean (SD)	24.8 (4.8)	26.0 (4.5)	24.6 (4.8)
	Physical activity level (inactive), n (%)	4381 (90.8)	775 (87.4)	3606 (91.6)
**Sleep status, mean (SD)**				
	Number of days of Pokémon Sleep recording	134.0 (54.5)	135.7 (56.0)	133.7 (54.2)
	Total sleep time (hours)	6.7 (1.1)	6.5 (1.1)	6.7 (1.1)
	Sleep onset latency (minutes)	21.5 (12.1)	23.5 (13.6)	21.1 (11.7)
	Percentage wakefulness after sleep onset (%)	9.9 (7.2)	13.1 (9.1)	9.2 (6.4)
**Nutrient status, mean (SD)**				
	Number of days of Asken data recording (days)	85.8 (63.6)	93.2 (67.3)	84.1 (62.7)
	Total energy intake (kcal)	1662.6 (330.3)	2055.1 (349.2)	1574.2 (252.4)
	Protein intake (% total kcal)	17.7 (3.7)	18.8 (4.8)	17.5 (3.4)
	Carbohydrate intake (% total kcal)	51.7 (5.5)	50.3 (5.9)	52.0 (5.3)
	Total fat intake (% total kcal)	32.3 (4.3)	31.4 (4.8)	32.6 (4.2)
	Saturated fat intake (% total kcal)	9.5 (1.8)	8.8 (1.8)	9.7 (1.8)
	Monounsaturated fat intake (% total kcal)	11.5 (2.0)	11.3 (2.2)	11.5 (1.9)
	Polyunsaturated fat intake (% total kcal)	6.3 (1.0)	6.1 (1.2)	6.3 (1.0)
	Dietary fiber intake (g/day)	11.7 (3.5)	10.7 (2.7)	11.9 (3.6)
	Sodium (mg/day)	3486.6 (881.0)	4230.7 (996.7)	3319.1 (758.1)
	Potassium (mg/day)	2295.3 (602.1)	2625.0 (692.4)	2221.0 (553.3)
	Sodium to Potassium ratio	1.6 (0.4)	1.7 (0.5)	1.6 (0.4)

The mean macronutrient composition was as follows: protein, 18.2%; carbohydrates, 53.8%; and total fat, 28% (saturated fat at 9.7%, monounsaturated fat at 11.8%, and polyunsaturated fat at 6.5%; Table S4 in Multimedia Appendix 1).

**Figure 2:**
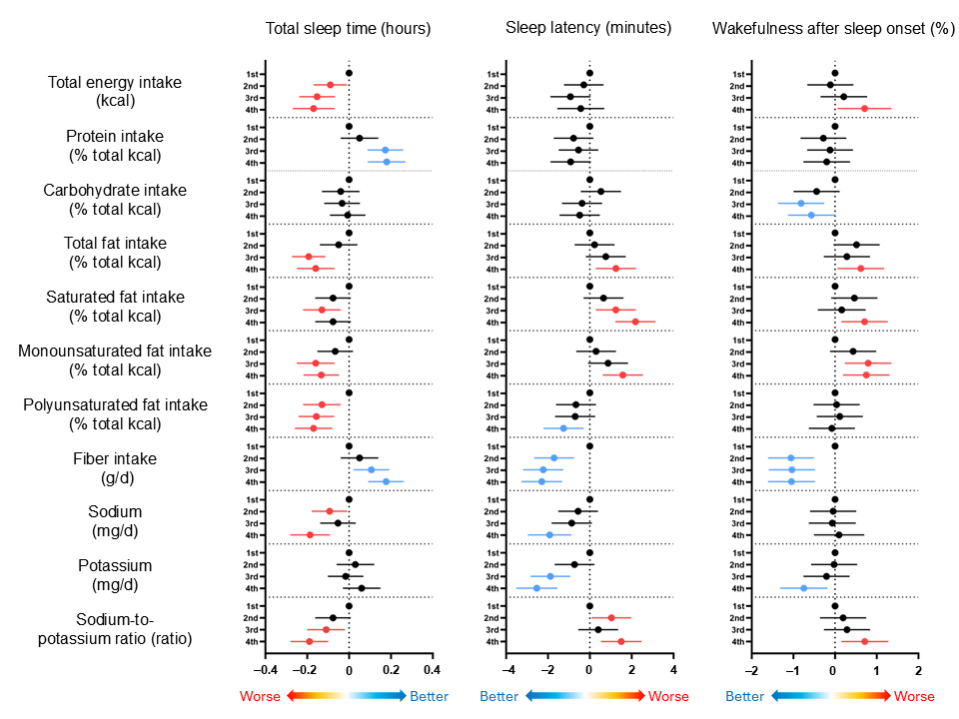
Multivariable regression analysis of macronutrients and dietary components on sleep variables.

Figure 2 and Table S5 in Multimedia Appendix 1 present the results of multivariate regression analyses comparing each nutrient across quartiles to the first quartile in relation to sleep variables. Specifically, in terms of macronutrients, the TST was significantly shorter in the second, third, and fourth quartiles compared with the first quartile with lower total energy intake. Greater intake ratios of total fat, including saturated, monounsaturated, and polyunsaturated fats, were associated with shorter TST. In contrast, greater protein intake levels in the third and fourth quartiles were associated with longer TST compared with those in the first quartile.

Regarding SL, greater levels of total, saturated, and monounsaturated fat intake were linked to longer SL, while greater intake of polyunsaturated fats showed a tendency toward shorter SL. Greater total energy and total fat intakes were associated with longer %WASO, whereas greater carbohydrate intake indicated a tendency towards a shorter %WASO. Greater dietary fiber intake consistently showed associations with longer TST, shorter SL, and shorter %WASO, suggesting its beneficial effect on sleep quality. Greater sodium intake was associated with shorter TST, and both greater sodium and potassium intake were linked to shorter SL. Notably, a greater sodium-to-potassium ratio was related to shorter TST, longer SL, and %WASO (Figure 2 and Table S5 in Multimedia Appendix 1).

**Figure 3:**
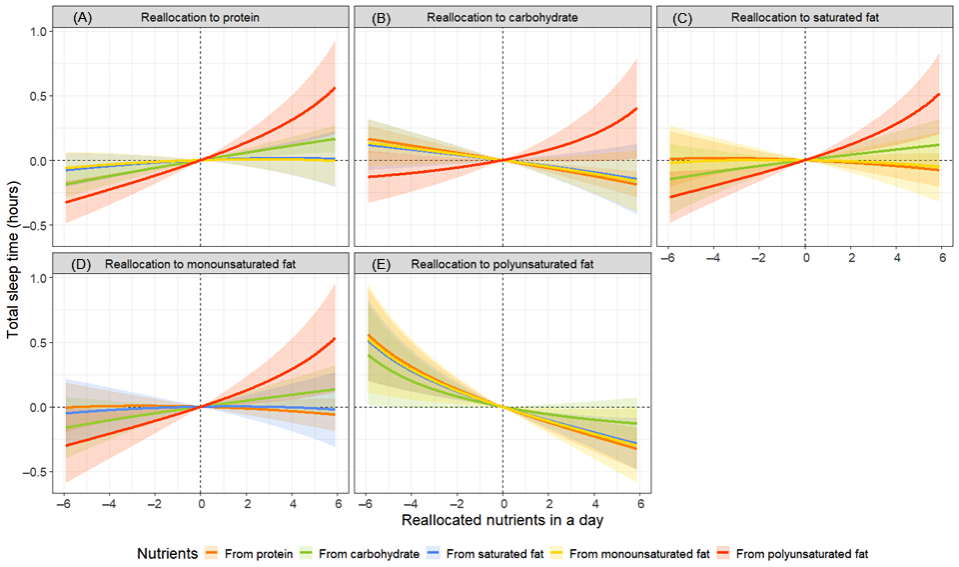
Simulated changes in total sleep time when reallocating fixed amounts of other nutrient components among each nutrient component: (A) protein, (B) carbohydrate, (C) saturated fat, (D) monounsaturated fat, and (E) polyunsaturated fat, while keeping the remaining components constant at compositional percentages. All estimates have been adjusted for age, sex, and BMI.

**Figure 4:**
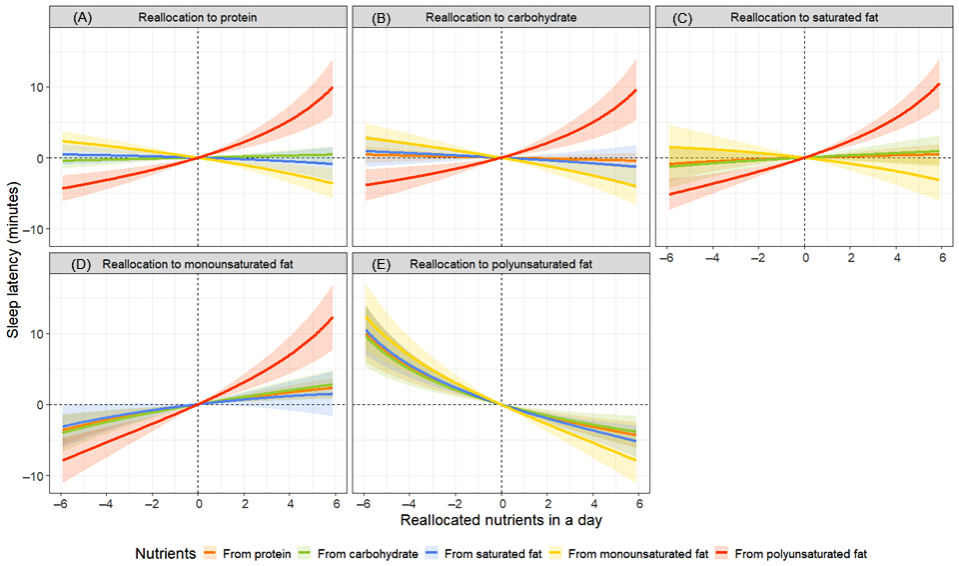
Simulated changes in sleep latency when reallocating fixed amounts of other nutrient components among each nutrient component: (A) protein, (B) carbohydrate, (C) saturated fat, (D) monounsaturated fat, and (E) polyunsaturated fat, while keeping the remaining components constant at compositional percentages. All estimates have been adjusted for age, sex, and BMI.

**Figure 5:**
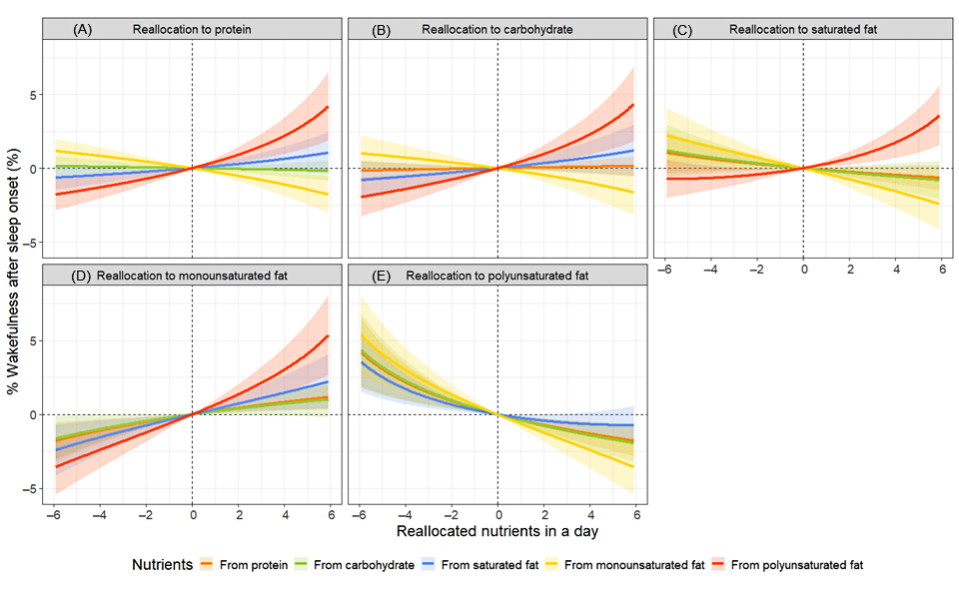
Simulated changes in the proportion of wakefulness after sleep onset when reallocating fixed amounts of other nutrient components among each nutrient component: (A) protein, (B) carbohydrate, (C) saturated fat, (D) monounsaturated fat, and (E) polyunsaturated fat, while keeping the remaining components constant at compositional percentages. All estimates have been adjusted for age, sex, and BMI.

The results of the isotemporal substitution model showed that replacing 6% of total energy intake from protein with carbohydrates was significantly associated with a reduction of 0.2 hours in TST. Conversely, replacing 6% of polyunsaturated fat with equivalent amounts of protein, carbohydrates, saturated fat, and monounsaturated fat was significantly associated with changes in the TST by +0.6, +0.4, +0.5, and +0.6 hours, respectively (Figure 3 and Table S6 in Multimedia Appendix 1). Regarding SL, replacing 6% of proteins or carbohydrates with monounsaturated fat was significantly associated with a change of +2.4 and +2.8 minutes, respectively. Conversely, replacing 6% of monounsaturated fat with equivalent amounts of protein, carbohydrates, and saturated fat was significantly associated with SL changes of –3.7, –4.1, and –3.2 minutes, respectively. Additionally, replacing 6% polyunsaturated fat with equivalent amounts of protein, carbohydrates, saturated fat, and monounsaturated fat was significantly associated with changes in SL of +10.3, +9.9, +10.8, and +12.7 minutes, respectively (Figure 4 and Table S6 in Multimedia Appendix 1). For %WASO, replacing 6% of protein with monounsaturated fat was significantly associated with a +1.2% change, whereas replacing saturated fat with monounsaturated fat was significantly associated with a +2.3% change. Conversely, replacing 6% of monounsaturated fat with equivalent amounts of protein, carbohydrates, and saturated fat was significantly associated with changes in %WASO by –1.8%, –1.7%, and –2.5%, respectively. Additionally, replacing 6% polyunsaturated fat with equivalent amounts of protein, carbohydrates, saturated fat, and monounsaturated fat was significantly associated with changes in %WASO of +4.3%, +4.5%, +3.7%, and +5.5%, respectively (Figure 5 and Table S6 in Multimedia Appendix 1).

In the compositional data analysis, changes in protein by –6% of the overall macronutrient composition were significantly associated with changes in the TST by –0.6 hours, whereas changes in polyunsaturated fat by –6% were significantly associated with changes in the TST by +0.5 hours. Conversely, changes in protein by +6% from the overall macronutrient composition were significantly associated with changes in TST by +0.3 hours, while changes in polyunsaturated fat by +6% were significantly associated with changes in TST by –0.2 hours (Table 2). Regarding SL, changes in monounsaturated fat by –6% from the overall macronutrient composition were significantly associated with a change of –10.5 minutes, whereas changes in polyunsaturated fat by –6% were associated with a change of +10.7 minutes. Conversely, changes in monounsaturated fat by +6% were significantly associated with a change of +4.6 minutes, and changes in polyunsaturated fat by +6% were significantly associated with a change of –4.7 minutes (Table 2). For %WASO, similar to SL, changes in monounsaturated fats by –6% of the overall macronutrient composition were significantly associated with a change of –5.0%, whereas a –6% change in polyunsaturated fats was significantly associated with a change of +4.5%. Conversely, changes in the monounsaturated fat content by +6% were significantly associated with a change of +2.2%, and changes in the polyunsaturated fat content by +6% were significantly associated with a change of –2.0% (Table 2).

**Table 2:** Estimated nonstandardized coefficients for displacing 6% of macronutrients between one and the remaining macronutrients proportionally. Data are presented as nonstandardized coefficients (95% CI). All the estimates were adjusted for age, sex, and BMI. Nonstandardized coefficients reflect the estimated change in sleep parameters associated with reallocating 6% of the macronutrients between one and the remaining macronutrients proportionally.

	Total sleep time (hours), 95% CI		Sleep latency (minutes), 95% CI			% Wakefulness after sleep onset (%), 95% CI	
	Take 6% from one nutrient and distribute it	Add 6% to one nutrient from the others	Take 6% from one nutrient and distribute it	Add 6% to one nutrient from the others	Take 6% from one nutrient and distribute it		Add 6% to one nutrient from the others
Protein	–0.61 (–0.80 to –0.41)^a^	+0.27 (0.18 to 0.35)^a^	–0.78 (–2.93 to 1.38)	+0.34 (–0.61 to 1.29)	–0.06 (–1.32 to 1.20)		+0.03 (–0.53 to 0.58)
Carbohydrate	+0.60 (–0.20 to 1.40)	–0.27 (–0.62 to 0.09)	+2.79 (–5.98 to 11.57)	–1.23 (–5.10 to 2.64)	–1.70 (–6.81 to 3.41)		+0.75 (–1.51 to 3.01)
Saturated fat	–0.20 (–0.67 to 0.28)	+0.09 (–0.12 to 0.30)	–2.20 (–7.40 to 2.99)	+0.97 (–1.32 to 3.26)	+2.23 (–0.80 to 5.26)		–0.99 (–2.32 to 0.35)
Monounsaturated fat	–0.30 (–0.85 to 0.26)	+0.13 (–0.11 to 0.38)	–10.50 (–16.63 to –4.38)^a^	+4.64 (1.93 to 7.34)^a^	–5.00 (–8.57 to –1.43)^a^		+2.21 (0.63 to 3.78)^a^
Polyunsaturated fat	+0.50 (0.12 to 0.88)^a^	–0.22 (–0.39 to –0.05)^a^	+10.69 (6.48 to 14.90)^a^	–4.72 (–6.58 to –2.86)^a^	+4.53 (2.07 to 6.99)^a^		–2.00 (–3.08 to –0.92)^a^

^a^Statistically significant (*P*<.05).

## Discussion

### Principal Findings

This study examined the relationship between nutritional intake and sleep using multivariate regression analysis, isotemporal substitution models, and compositional data analysis. Overall, the findings are consistent with those of previous research on individual nutrients and sleep [3-6,16-21]. As shown in Figure 2, greater total energy, fat, and sodium intakes were associated with shorter TST, whereas greater protein and dietary fiber intakes were linked to longer TST. Furthermore, the main findings of this study, based on compositional data analysis considering interdependencies, demonstrated that increasing protein while reducing other factors is associated with an elevated TST, whereas an increase in the polyunsaturated fat with a reduced TST (Table 2). Regarding SL and WASO, monounsaturated fat and polyunsaturated fat exhibited opposing relationships (Table 2). The differences in the compositional data analysis results, compared with individual macronutrients (eg, carbohydrates), likely reflect the strong interdependent relationships among various nutrients.

Dietary fiber intake was consistently associated with improved SL and %WASO. Additionally, greater sodium intake was correlated with shorter TST, whereas greater potassium intake was associated with shorter SL. Notably, polyunsaturated fats demonstrated potential benefits for SL and %WASO compared with other types of fat. Studies suggest that dietary fiber can affect sleep by influencing microbiota [39,40]. When dietary fibers are fermented by microbiota in the large intestine, they produce short-chain fatty acids, such as acetate, propionate, and butyrate, which enhance serotonin release. Furthermore, administering tributyrin, a compound made of three molecules of butyric acid and glycerol, to mice resulted in a 50% increase in nonrapid eye movement sleep for 4 h [40]. The results of this study support those of previous studies [39,40].

Previous studies have established that greater total energy intake and poor dietary quality are associated with lower subjective sleep quality [7,41]. Specifically, higher protein intake correlates with improved sleep, whereas a higher intake of fatty acids is linked to worse sleep [41]. This study also found variations in the effects of lipid intake on sleep, depending on the type (eg, saturated, monounsaturated, or polyunsaturated fats). The mechanism by which proteins affect sleep following acute feeding may involve tryptophan; tyrosine; and the synthesis of brain neurotransmitters, such as serotonin, melatonin, and dopamine [42]. Although no significant differences were observed in SL or %WASO, a greater protein intake was positively associated with longer TST.

However, the specific mechanisms underlying the relationship between monounsaturated fat, polyunsaturated fat, and sleep remain unclear. A meta-analysis found an association between a greater intake of hexadecenoic acid, a saturated fatty acid, and subjective difficulty falling asleep [43]. Conversely, difficulties falling asleep were linked to a lower intake of dodecanoic acid, a monounsaturated fat, and butanoic acid, a saturated fatty acid [43]. Additionally, a reduced intake of dodecanoic acid was associated with subjective difficulties in maintaining sleep [43]. An epidemiological study involving adults evaluated the relationship between two polyunsaturated fatty acids, ω-6 and ω-3, and found that ω-6 fatty acids were related to the risk of sleep disorder, based on self-reported data, in an inverse U-shaped manner [19]. The ω-6:ω-3 ratio was positively and linearly associated with the risk of sleep disorders [19]. The underlying mechanisms involving ω-3 fatty acids and sleep are discussed, noting that a deficiency in ω-3 intake may affect cortical neuron oscillatory activity and sleep-wake patterns [20].

Additionally, the timing of fat intake may influence sleep; one observational study based on polysomnography suggested that consuming high-fat foods close to bedtime may be associated with increased WASO [44]. Continued consumption of a high-fat diet has been partially understood to reduce physical activity and increase rapid eye movements and sleep fragmentation through dopaminergic dysregulation [45]. Research involving mouse models and pregnant women has suggested a potential positive association between monounsaturated fat intake and sleep [46,47]. However, other studies have indicated a negative association between monounsaturated fat and sleep, whereas polyunsaturated fat has shown a positive association with sleep [48-50]. Previous studies have often focused on dietary fiber levels and comparisons between high- and low-saturated-fat diets [45,48]. Future studies should explore the impact of unsaturated fats, including monounsaturated and polyunsaturated fats.

Sodium and potassium have been reported to impact health individually [51,52], and a high sodium-to-potassium ratio (indicating relatively high sodium intake) has been associated with higher all-cause mortality [53]. Sodium intake is known to be correlated with sleep, indicating that greater sodium intake is associated with shorter subjective TST and worse sleep quality [30,50]. This relationship is attributable to the potential effect of sodium on nocturnal urination [51,52]. However, in this study, a greater intake of sodium alone was associated with a shorter TST, although no significant differences were observed in %WASO. Moreover, greater sodium intake was associated with shorter SL. Greater potassium intake was associated with shorter SL and reduced %WASO. All sleep variables were adversely related when considering the balance between sodium and potassium, which indicates an unhealthy dietary pattern (ie, relatively greater sodium intake). In this study, sodium intake was used as a basis for analysis, while the sodium-to-potassium ratio was also evaluated (Figure 2). Therefore, the results linking sleep outcomes with the sodium-to-potassium ratio are considered reasonable, as they provide a more comprehensive perspective on the impact of both minerals on sleep.

Recent studies suggested that changes in the gut microbiota may influence sleep and nutrient absorption [39,54,55]. Disrupted gut flora in patients with celiac disease has been linked to sleep disturbances [56]. Future research is warranted especially focusing on patients with celiac disease. Additionally, digital health tools offer the potential for health monitoring and early diagnosis [57].

### Limitations

In this study, we investigated the association between daily nutrient intake and sleep using isotemporal substitution models and compositional data analyses to account for the interdependencies [25]. These approaches enabled us to assess how substituting macronutrients and considering the overall composition ratio affect sleep outcomes. Nonetheless, this study had several limitations. First, this cross-sectional study does not provide causal explanations. As noted above, previous studies have reported bidirectional relationships between these factors [58]. Additionally, the isotemporal substitution model and compositional data analysis are merely statistical inferences [25]. The findings of this study underscore the need for future interventions and longitudinal studies to establish causal effects definitively.

Second, this study may have included a highly health-conscious group that uses both health management apps simultaneously. Therefore, although values around the second and third quartiles, as shown in Table S1 in Multimedia Appendix 1, correspond to Japanese macronutrient standards [59] and suggest the possibility of representing the general population, caution is needed when generalizing the findings to broader populations, owing to the inclusion of this health-conscious group.

Third, the sleep assessment method used in this study involved applying gamified elements. Some users may have exhibited sleep patterns different from their habitual patterns to achieve specific goals within the game. Moreover, because users have their own smartphones, the reliability of accelerometer measurements varies across different device models. In the future, integrating alternative methods, such as tappigraphy for sleep-wake rhythms, actigraphy, or in-home electroencephalogram devices could provide more objective evaluations [13-15].

Fourth, retrospective studies like this one are inherently prone to recall bias, as participants may inaccurately report their dietary intake and sleep patterns based on memory. It should also be noted that dietary intake was not cross-checked, and this study did not separately account for weekdays and weekends in assessing overall nutrient intake. Finally, because this study was retrospective, we could not adequately capture confounding factors related to sleep and nutrition. We collected basic demographic information, such as age, sex, height, weight, and physical activity level. However, other confounding factors, such as alcohol and smoking habits, employment status (including shifts and night shifts), marital status, cohabitation status, medical history, and medication use, were not considered. Therefore, since we could not fully eliminate concerns regarding internal validity, the presence of selection bias cannot be ruled out. Although this is an observational study with a large sample size of real-world data, it is essential to exercise caution in interpreting the results owing to these limitations.

### Conclusions

We found that a greater intake of polyunsaturated fats was associated with better SL and reduced WASO, while saturated and monounsaturated fats were correlated with longer SL and wakefulness. Dietary fiber intake was positively associated with improved sleep quality, and a greater sodium-to-potassium ratio was linked to worse sleep outcomes. Furthermore, our compositional data analysis, which accounted for the interdependencies between nutrients, suggested that increasing the protein intake in place of other nutrients could improve the TST, and similar effects were observed for polyunsaturated fats on SL and WASO. Although this study is cross-sectional, the results highlight the complex potential role of dietary factors in sleep regulation and suggest the possibility of dietary interventions to enhance sleep health.

## Appendix

Multimedia Appendix 1 Supplementary Information.

## Abbreviations

%WASO: percentage of wakefulness after sleep onset
ilr: isometric log-ratio
SL: sleep latency
TST: total sleep time
WASO: wakefulness after sleep onset

## Data Availability

The data used in this study belong to The Pokémon Company (Tokyo, Japan) and Asken Inc (Tokyo, Japan). These data are not publicly available but can be used in future projects. Proposals and requests for data access were sent to the corresponding authors via email.
