# Supplementary material for: Relationship Among Macronutrients, Dietary Components, and Objective Sleep Variables Measured by Smartphone Apps: Real-World Cross-Sectional Study
Source: J Med Internet Res. 2025 Jan 30;27:e64749. doi: 10.2196/64749 (PMC11826953; doi:10.2196/64749)
Supplement: Multimedia Appendix 1 [file jmir_v27i1e64749_app1.docx]

**Supplementary Information**

**Table S1**. Range of quartiles for macronutrient and dietary components in multivariable regression analysis

|  | 1^st^ quartile  (n = 1207) | 2^nd^ quartile  (n = 1206) | 3^rd^ quartile  (n = 1206) | 4^th^ quartile  (n = 1206) |
| --- | --- | --- | --- | --- |
| Total energy intake  (kcal) | 369.13–1450.56 | 1450.66–1619.27 | 1619.32–1834.00 | 1834.04–5019.60 |
| Protein intake  (% total kcal) | 8.31–15.24 | 15.25–16.86 | 16.87–19.27 | 19.28–48.37 |
| Carbohydrate intake  (% total kcal) | 14.14–48.74 | 48.75–52.08 | 52.08–55.12 | 55.12–84.84 |
| Total fat intake  (% total kcal) | 15.29–29.68 | 29.69–32.43 | 32.44–35.01 | 35.02–61.33 |
| Saturated fat intake  (% total kcal) | 2.34–8.31 | 8.32–9.48 | 9.48–10.67 | 10.67–19.29 |
| Monounsaturated fat intake  (% total kcal) | 2.65–10.28 | 10.29–11.54 | 11.54–12.74 | 12.74–24.64 |
| Polyunsaturated fat intake  (% total kcal) | 1.34–5.63 | 5.63–6.25 | 6.26–6.89 | 6.89–13.61 |
| Dietary fiber intake  (g/d) | 2.65–9.61 | 9.62–11.04 | 11.05–12.85 | 12.85–104.21 |
| Sodium  (mg/d) | 406.80–2918.86 | 2919.01–3405.71 | 3406.36–3959.13 | 3959.18–11506.16 |
| Potassium  (mg/d) | 333.31–1889.13 | 1889.45–2232.65 | 2232.71–2635.82 | 2636.15–6133.66 |
| Sodium to Potassium ratio (ratio) | 0.19–1.30 | 1.30–1.54 | 1.55–1.81 | 1.81–4.87 |

**Table S2.** Compositional variation matrix of macronutrient components

| Variables | Protein | Carbohydrate | Saturated fat | Monounsaturated fat | Polyunsaturated fat |
| --- | --- | --- | --- | --- | --- |
| Protein | 0 | 0.064 | 0.101 | 0.085 | 0.064 |
| Carbohydrate | 0.064 | 0 | 0.013 | 0.008 | 0.019 |
| Saturated fat | 0.101 | 0.013 | 0 | 0.016 | 0.047 |
| Monounsaturated fat | 0.085 | 0.008 | 0.016 | 0 | 0.023 |
| Polyunsaturated fat | 0.064 | 0.019 | 0.047 | 0.023 | 0 |

Note: A value close to zero implies that the intake of the two macronutrient components involved in the ratio is highly proportional.

**Table S3.** Association of macronutrient components with sleep parameters in study participants

|  | Total sleep time (hours) | | Sleep latency (minutes) | | % Wakefulness after sleep onset (%) | |
| --- | --- | --- | --- | --- | --- | --- |
| Variables | B | (95%CI) | B | (95%CI) | B | (95%CI) |
| Protein  (% total kcal) | **0.48** | **(0.33, 0.64)** | 0.62 | (–1.10, 2.33) | 0.05 | (–0.95, 1.05) |
| Carbohydrate  (% total kcal) | –0.48 | (–1.11, 0.16) | -2.22 | (–9.19, 4.75) | -1.48 | (–2.71, 5.41) |
| Saturated fat  (% total kcal) | 0.16 | (–0.22, 0.53) | 1.75 | (–2.38, 5.88) | -1.77 | (–4.18, 0.63) |
| Monounsaturated fat  (% total kcal) | 0.24 | (–0.21, 0.68) | **8.35** | **(3.48, 13.21)** | **3.97** | **(1.14, 6.81)** |
| Polyunsaturated fat  (% total kcal) | **–0.40** | **(–0.70, –0.09)** | **–8.49** | **(–11.84, –5.15)** | **–3.60** | **(–5.55, –1.65)** |

Note: Data are presented as non-standardized coefficients with 95% confidence intervals. The results in bold are significant (*P* < .05). All estimates have been adjusted for age, sex, body mass index.

|  | Mean | Min | Max |
| --- | --- | --- | --- |
| Protein (% total kcal) | 18.2 | 8.5 | 45.4 |
| Carbohydrate (% total kcal) | 53.8 | 15.1 | 76.9 |
| Saturated fat (% total kcal) | 9.7 | 2.8 | 20.5 |
| Monounsaturated fat (% total kcal) | 11.8 | 3.1 | 26.2 |
| Polyunsaturated fat (% total kcal) | 6.5 | 1.7 | 13.8 |

**Table S4**. Compositional ratios of macronutrients among participants

|  |  | Total sleep time (hour) | | Sleep latency (min) | | % Wakefulness after sleep onset (%) | |
| --- | --- | --- | --- | --- | --- | --- | --- |
|  |  | Unadjusted | Sex, age, and BMI adjusted | Unadjusted | Sex, age, and BMI adjusted | Unadjusted | Sex, age, and BMI adjusted |
| Total energy intake (kcal) | 2^nd^ quartile | **–0.09 (–0.18, –0.01)** | **–0.09 (–0.17, –0.01)** | –0.19 (–1.15, 0.78) | –0.29 (–1.24, 0.66) | –0.01 (–0.57, 0.56) | –0.11 (–0.67, 0.44) |
|  | 3^rd^ quartile | **–0.18 (–0.26, –0.09)** | **–0.15 (–0.24, –0.07)** | –0.38 (–1.34, 0.59) | –0.93 (–1.89, 0.03) | **0.73 (0.17, 1.29)** | 0.21 (–0.35, 0.77) |
|  | 4^th^ quartile | **–0.23 (–0.32, –0.15)** | **–0.17 (–0.27, –0.07)** | **1.55 (0.58, 2.51)** | –0.43 (–1.56, 0.69) | **2.88 (2.32, 3.44)** | **0.71 (0.06, 1.36)** |
| Protein intake  (% total kcal) | 2^nd^ quartile | 0.04 (–0.05, 0.13) | 0.05 (–0.04, 0.14) | –0.69 (–1.65, 0.28) | –0.78 (–1.73, 0.17) | –0.20 (–0.77, 0.38) | –0.28 (–0.83, 0.27) |
|  | 3^rd^ quartile | **0.17 (0.08, 0.26)** | **0.17 (0.09, 0.26)** | –0.38 (–1.35, 0.58) | –0.54 (–1.49, 0.41) | –0.05 (–0.62, 0.52) | –0.12 (–0.67, 0.43) |
|  | 4^th^ quartile | **0.17 (0.08, 0.25)** | **0.18 (0.09, 0.27)** | –0.09 (–1.06, 0.87) | –0.92 (–1.88, 0.05) | 0.34 (–0.24, 0.91) | –0.20 (–0.76, 0.36) |
| Carbohydrate　intake  (% total kcal) | 2^nd^ quartile | –0.01 (–0.10, 0.08) | –0.04 (–0.13, 0.05) | 0.27 (–0.70, 1.24) | 0.53 (–0.43, 1.49) | **–0.89 (–1.46, –0.32)** | –0.44 (–1.00, 0.11) |
|  | 3^rd^ quartile | 0.01 (–0.08, 0.09) | –0.03 (–0.12, 0.05) | –0.75 (–1.71, 0.22) | –0.37 (–1.33, 0.59) | **–1.35 (–1.92, –0.78)** | **–0.82 (–1.37, –0.26)** |
|  | 4^th^ quartile | 0.05 (–0.04, 0.13) | –0.01 (–0.09, 0.08) | **–1.21 (–2.18, –0.25)** | –0.49 (–1.45, 0.48) | **–1.41 (–1.98, –0.84)** | **–0.57 (–1.13, –0.01)** |
| Total fat intake  (% total kcal) | 2^nd^ quartile | –0.06 (–0.15, 0.03) | –0.05 (–0.14, 0.04) | 0.03 (–0.65, 1.28) | 0.23 (–0.73, 1.18) | 0.54 (–0.03, 1.11) | 0.52 (–0.04, 1.07) |
|  | 3^rd^ quartile | **–0.11 (–0.20, –0.24)** | **–0.11 (–0.20, –0.27)** | 0.93 (–0.04, 1.89) | 0.76 (–0.19, 1.72) | 0.17 (–0.40, 0.74) | 0.28 (–0.27, 0.84) |
|  | 4^th^ quartile | **–0.16 (–0.25, –0.08)** | **–0.16 (–0.25, –0.07)** | **1.60 (0.63, 2.56)** | **1.25 (0.28, 2.21)** | **0.62 (0.05, 1.19)** | **0.62 (0.06, 1.18)** |
| Saturated fat intake  (% total kcal) | 2^nd^ quartile | –0.07 (–0.16, 0.02) | –0.08 (–0.16, 0.01) | 0.54 (–0.43, 1.50) | 0.65 (–0.30, 1.61) | 0.23 (–0.34, 0.80) | 0.46 (–0.09, 1.02) |
|  | 3^rd^ quartile | **–0.11 (–0.20, –0.02)** | **–0.13 (–0.22, –0.04)** | **1.16 (0.19, 2.12)** | **1.24 (0.29, 2.20)** | –0.25 (–0.82, 0.32) | 0.15 (–0.41, 0.74) |
|  | 4^th^ quartile | –0.05 (–0.14, 0.04) | –0.08 (–0.16, 0.01) | **2.04 (1.08, 3.01)** | **2.18 (1.22, 3.14)** | 0.19 (–0.38, 0.77) | **0.71 (0.15, 1.27)** |
| Monounsaturated fat intake  (% total kcal) | 2^nd^ quartile | –0.07 (–0.16, 0.02) | –0.07 (–0.15, 0.02) | 0.48 (–0.49, 1.44) | 0.30 (–0.65, 1.25) | 0.48 (–0.09, 1.05) | 0.43 (–0.12, 0.99) |
|  | 3^rd^ quartile | **–0.16 (–0.25, –0.08)** | **–0.16 (–0.25, –0.07)** | **1.13 (0.17, 2.10)** | 0.87 (–0.08, 1.83) | **0.84 (0.27, 1.41)** | **0.79 (0.24, 1.35)** |
|  | 4^th^ quartile | **–0.14 (–0.23, –0.06)** | **–0.13 (–0.22, –0.05)** | **2.22 (1.25, 3.18)** | **1.58 (0.62, 2.54)** | **0.96 (0.39, 1.53)** | **0.75 (0.19, 1.31)** |
| Polyunsaturated fat intake  (% total kcal) | 2^nd^ quartile | **–0.13 (–0.22, –0.05)** | **–0.13 (–0.22, –0.04)** | –0.68 (–1.65, 0.29) | –0.66 (–1.62, 0.29) | –0.05 (–0.62, 0.52) | 0.04 (–0.52, 0.59) |
|  | 3^rd^ quartile | **–0.16 (–0.25, –0.07)** | **–0.16 (–0.24, –0.07)** | –0.62 (–1.59, 0.35) | –0.70 (–1.66, 0.26) | 0.04 (–0.53, 0.61) | 0.11 (–0.44, 0.67) |
|  | 4^th^ quartile | **–0.18 (–0.27, –0.09)** | **–0.17 (–0.26, –0.08)** | **–1.22 (–2.19, –0.25)** | **–1.26 (–2.22, –0.30)** | –0.13 (–0.70, 0.44) | –0.08 (–0.63, 0.48) |
| Dietary fiber intake (g/d) | 2^nd^ quartile | 0.06 (–0.03, 0.15) | 0.05 (–0.04, 0.14) | **–1.99 (–2.96, –1.03)** | **–1.71 (–2.66, –0.76)** | **–1.38 (–1.94, –0.81)** | **–1.06 (–1.61, –0.51)** |
|  | 3^rd^ quartile | **0.12 (0.03, 0.21)** | **0.11 (0.02, 0.19)** | **–2.88 (–3.85, –1.92)** | **–2.23 (–3.19, –1.27)** | **–1.65 (–2.21, –1.08)** | **–1.04 (–1.59, –0.48)** |
|  | 4^th^ quartile | **0.19 (0.11, 0.28)** | **0.18 (0.09, 0.26)** | **–3.21 (–4.17, –2.25)** | **–2.30 (–3.27, –1.33)** | **–1.84 (–2.41, –1.27)** | **–1.05 (–1.61, –0.48)** |
| Sodium (mg/d) | 2^nd^ quartile | **–0.10 (–0.19, –0.02)** | **–0.09 (–0.18, –0.01)** | –0.10 (–1.07, 0.86) | –0.56 (–1.51, 0.39) | 0.27 (–0.29, 0.84) | –0.05 (–0.60, 0.51) |
|  | 3^rd^ quartile | –0.08 (–0.17, 0.01) | –0.05 (–0.14, 0.03) | 0.01 (–0.96, 0.98) | –0.87 (–1.83, 0.10) | **0.68 (0.11, 1.24)** | –0.07 (–0.63, 0.50) |
|  | 4^th^ quartile | **–0.25 (–0.34, –0.16)** | **–0.19 (–0.28, –0.09)** | 0.10 (–0.87, 1.07) | **–1.92 (–2.96, –0.88)** | **2.06 (1.50, 2.63)** | 0.10 (–0.51, 0.70) |
| Potassium (mg/d) | 2^nd^ quartile | 0.02 (–0.07, 0.10) | 0.03 (–0.06, 0.12) | –0.52 (–1.48, 0.45) | –0.73 (–1.68, 0.22) | 0.23 (–0.35, 0.80) | –0.02 (–0.58, 0.53) |
|  | 3^rd^ quartile | –0.05 (–0.13, 0.04) | –0.02 (–0.10, 0.07) | **–1.61 (–2.57, –0.64)** | **–1.89 (–2.84, –0.93)** | 0.27 (–0.30, 0.84) | –0.21 (–0.76, 0.35) |
|  | 4^th^ quartile | –0.01 (–0.09, 0.08) | 0.06 (–0.03, 0.15) | **–1.82 (–2.78, –0.85)** | **–2.54 (–3.52, –1.56)** | 0.42 (–0.15, 1.00) | **–0.75 (–1.32, –0.18)** |
| Sodium to Potassium ratio (ratio) | 2^nd^ quartile | **–0.09 (–0.18, –0.01)** | –0.08 (–0.16, 0.01) | **1.30 (0.33, 2.26)** | **1.03 (0.08, 1.98)** | 0.41 (–0.16, 0.98) | 0.19 (–0.36, 0.75) |
|  | 3^rd^ quartile | **–0.12 (–0.21, –0.04)** | **–0.11 (–0.20, –0.02)** | **0.99 (0.03, 1.95)** | 0.40 (–0.55, 1.36) | **0.75 (0.18, 1.32)** | 0.29 (–0.27, 0.84) |
|  | 4^th^ quartile | **–0.21 (–0.29, –0.12)** | **–0.19 (–0.28, –0.10)** | **2.53 (1.57, 3.50)** | **1.50 (0.53, 2.47)** | **1.45 (0.88, 2.02)** | **0.71 (0.15, 1.28)** |

**Table S5**. Multivariable regression analysis of macronutrients and dietary components on sleep variables

Note: Data are presented as non-standardized coefficients with 95% confidence intervals. The results in bold are significant (*P* < .05).

**Table S6**. Changes in sleep parameters when reallocating fixed amounts of other nutrient components among each nutrient component, while keeping the remaining components constant at compositional percentages.

| ±6% | To protein | To carbohydrate | To saturated fat | To monounsaturated fat | To polyunsaturated fat |
| --- | --- | --- | --- | --- | --- |
| **Total sleep time (hours)** | | | | | |
| From protein | Drop | **–0.19**  **(–0.29, –0.09)** | –0.08  (–0.21, 0.05) | –0.06  (–0.19, 0.07) | **–0.33**  **(–0.50, –0.17)** |
| From carbohydrate | **+0.17**  **(0.06, 0.27)** | Drop | +0.12  (–0.08, 0.32) | +0.14  (–0.05, 0.32) | –0.13  (–0.34, 0.07) |
| From saturated fat | +0.01  (–0.21, 0.23) | –0.15  (–0.43, 0.13) | Drop | –0.02  (–0.32, 0.27) | **–0.29**  **(–0.49, –0.09)** |
| From monounsaturated fat | –0.01  (–0.20, 0.19) | –0.17  (–0.41, 0.08) | –0.05  (–0.33, 0.22) | Drop | **–0.31**  **(–0.60, –0.01)** |
| From polyunsaturated fat | **+0.58**  **(0.21, 0.96)** | **+0.42**  **(0.02, 0.83)** | **+0.53**  **(0.21, 0.86)** | **+0.55**  **(0.12, 0.98)** | Drop |
| **Sleep latency (minutes)** | | | | | |
| From protein | Drop | –0.44  (–1.56, 0.69) | +0.45  (–1.00, 1.91) | **+2.35**  **(0.94, 3.76)** | **–4.38**  **(–6.20, –2.55)** |
| From carbohydrate | +0.44  (–0.73, 1.60) | Drop | +0.93 (–1.29, 3.14) | **+2.82**  **(0.77, 4.88)** | **–3.90**  **(–6.15, –1.65)** |
| From saturated fat | –0.92  (–3.35, 1.51) | –1.31  (–4.39, 1.77) | Drop | +1.47  (–1.76, 4.70) | **–5.25**  **(–7.49, –3.01)** |
| From monounsaturated fat | **–3.70**  **(–5.85, –1.54)** | **–4.09**  **(–6.78, –1.41)** | **–3.21**  **(–6.22, –0.19)** | Drop | **–8.03**  **(–11.27, –4.79)** |
| From polyunsaturated fat | **+10.34**  **(6.22, 14.46)** | **+9.94**  **(5.49, 14.40)** | **+10.83**  **(7.25, 14.41)** | **+12.73**  **(7.96, 17.50)** | Drop |
| **% Wakefulness after sleep onset (%)** | | | | | |
| From protein | Drop | +0.15  (–0.51, 0.80) | –0.64  (–1.49, 0.20) | **+1.19**  **(0.36, 2.01)** | **–1.80**  **(–2.86, –0.73)** |
| From carbohydrate | –0.18  (–0.85, 0.50) | Drop | –0.82  (–2.11, 0.48) | +1.01  (–0.19, 2.21) | **–1.97**  **(–3.28, –0.66)** |
| From saturated fat | +1.07  (–0.34, 2.49) | +1.22  (–0.57, 3.02) | Drop | **+2.26**  **(0.38, 4.14)** | –0.72  (–2.02, 0.59) |
| From monounsaturated fat | **–1.81**  **(–3.07, –0.56)** | **–1.66**  **(–3.23, –0.10)** | **–2.45**  **(–4.21, –0.70)** | Drop | **–3.60**  **(–5.49, –1.72)** |
| From polyunsaturated fat | **+4.34**  **(1.93, 6.74)** | **+4.49**  **(1.89, 7.08)** | **+3.70**  **(1.61, 5.78)** | **+5.52**  **(2.74, 8.31)** | Drop |

Note: Data are presented as non-standardized coefficients with 95% confidence intervals. The results in bold are significant (*P* < .05). All estimates have been adjusted for age, sex, body mass index.
